# ‘What about the coffee break?’: Designing virtual conference spaces for conviviality

**DOI:** 10.1002/geo2.114

**Published:** 2022-11-09

**Authors:** Michelle Bastian, Emil Flatø, Lisa Baraitser, Helge Jordheim, Laura Salisbury, Thom van Dooren

**Affiliations:** University of Edinburgh; University of Oslo; Birkbeck; University of Oslo; Exeter University; University of Sydney

**Keywords:** online conferences, virtual meetings, time studies, flying less, climate change

## Abstract

Geography, like many other disciplines, is reckoning with the carbon intensity of its practices and rethinking how activities such as annual meetings are held. The Climate Action Task Force of the American Association of Geographers (AAG), for example, was set up in 2019 and seeks to transform the annual conference in light of environmental justice concerns. Mirroring shifts it geographic practice across the globe, these efforts point to a need to understand how new opportunities for knowledge production such as online events can operate effectively. In this article, we offer suggestions for best practice in virtual spaces arising from our Material Life of Time conference held in March 2021, a two day global event that ran synchronously across 15 time zones. Given concerns about lack of opportunities for informal exchanges at virtual conferences, or the “coffee break problem”, we designed the event to focus particularly on opportunities for conviviality. This was accomplished through a focus on three key design issues: the spatial, the temporal and the social. We review previous work on the benefits and drawbacks of synchronous and asynchronous online conference methods and the kinds of geographic communities they might support. We then describe our design approach and reflect on its effectiveness via a variety of feedback materials. We show that our design enabled high delegate satisfaction, a sense of conviviality, and strong connections with new colleagues. However we also discuss the problems with attendance levels and external commitments which hampered shared time together. We thus call for collective efforts to support the ‘event time’ of online meetings, rather than expectations to fit them around everyday tasks. Even so, our results suggest that synchronous online events need not result in geographical exclusions linked to time zone differences, and we outline further recommendations for reworking the spacetimes of the conference.

## 1 Introduction

For geographers, and indeed the academy in general, the online conference has become many things: a reluctantly accepted necessity during pandemic lockdowns and travel restrictions; a hoped-for change to carbon intensive academic practices in light of the climate emergency; an opportunity to challenge exclusions on multiple fronts (e.g. Hopkins et al., 2019; Jepson et al., 2022; Martin & Nevins, 2022). Mixed feelings are common – regret, anxiety, suspicion – but also delighted anticipation of what these new formats might offer. In this article, we reflect on The Material Life of Time, a synchronous online conference that first took shape in 2019 as a response to the climate crisis and took place in March 2021 over a year into the Covid-19 pandemic. The event was organised by the Temporal Belongings research network, a group of scholars working on social aspects of time, in collaboration with the Lifetimes project at the University of Oslo; the Waiting Times research group at Birkbeck, University of London and University of Exeter in the UK; and the Sydney Environment Institute at the University of Sydney.

Originally an international and in-person series, the Temporal Belongings conference moved online in an effort to respond to calls to reimagine how and why academics meet, in part, by meeting digitally. Anthropologist Hannah Knox, for example, has pointed to the growing realisation amongst scholars of the environment that “the very way in which they made knowledge was part of the cause of climate change” (2022, p. 163), thus pushing us to reconsider the material impacts of knowledge production. And much like geographer Sue Ruddick, we wanted to be part of working out how “to replace old seductions with new ones, story the things we value differently, reimagine infrastructures” (2019, p.577). At the same time, though, we were interested in how might we address concerns about a lack of serendipitous encounters, freedom of movement and coffee break chatter that dog the full acceptance of virtuality. As STS scholar Anne Pasek has argued “a shift to the digital must further imply a shift within the social if such transitions are to be successful” (2020, p36). We thus offer The Material Life of Time conference as a case study for how to make *convivial* online events.^1^ We reflect on two interconnected areas of our event design that speak particularly to conviviality, namely: our interest in creating an “event time” that could produce a sense of co-presence in the virtual space; and our efforts to enable attendees to interact autonomously and creatively in the space, alongside offering some of the joyful activities that have largely been associated with meeting in-person.

There is a burgeoning literature on online conferencing, a literature that has only increased with the significant number of events becoming virtual during the pandemic (see Rubinger et al., 2020 and Spilker et al., 2020). Given our focus we review some of what has been written so far about the social aspects of online events and the key issues that have arisen. We then focus our discussion on what we did, particularly the three areas that we concentrated our planning around: temporal design, spatial design and social design. This includes how we organised a synchronous international event that paid attention to disparities of time zones; designed a conference space that was intuitive, provided freedom of movement, and gave a sense of “place”; and how we responded to the perennial question of “what about the coffee breaks?” We then share what worked well and what did not, using a range of feedback materials including, most prominently, a post-conference survey of participants.

Overall, we argue that the labour of community building is brought front and centre with the move to online. The labour of hosting is often erased at academic events, no matter the type – yet it is an activity which is essential to their success. At physical conferences the tasks of welcoming, making comfortable, orienting and making sure that attendees feel ‘seen’, is often relegated to the volunteers on the registration desk, the staff at the conference centre, or the early career researchers who may have been drafted in to provide free support. By contrast, we argue that those invested in building new traditions of online events must think deliberately about how our ‘being together’ is facilitated and nurtured. This is particularly the case after the rush to move events online due the pandemic – where tight time frames and huge logistical challenges – made getting any alternative up and running more crucial than thinking about what might happen outside of, or around, the presentations themselves. Overall, we argue that paying at least as much attention to hosting and community-building, as to programme design and delivery, is vital for embedding online conferences as activities that people want to go to. At the same time, we raise concerns about the apparent norm, already being rapidly entrenched, that online conferences are not seen as ‘events’ that we can prioritise in our schedules and block off from other commitments, thus leaving little time for attending regular sessions, let alone all the activities that might make these events convivial.

## 2 Being together in time

As a group of interdisciplinary scholars who work on the social and philosophical dimensions of time, we were fascinated by how often the different temporal affordances of online formats are thematised in considerations of online sociality. Experiments with digital conferences bring participants together in a variety of ways, leading to diverse strategies for social interaction that do not always rely on co-presence. On the face of it, online formats do not require the condensed time frame of a conference where an international network descends on a city for a few days. Several conference formats have thus seized the opportunity to innovate “extemporaneous” or “asynchronous” meeting formats (Boon & Sleigh, 2020; Hiltner, 2016; Houghton et al., 2020). The Nearly Carbon Neutral (NCN) format (Hiltner, 2016), for example, provides a web interface with pre-recorded talks and space for written Q&A discussion underneath. Hiltner likens this approach to a form of social media, emphasising the ability of attendees to interact asynchronously with the content and each other, while capitalising on the way social media platforms have normalised these forms of sociality within the online context.

The case for asynchronous conferences, explored particularly well in a paper by Carr and Ludvigsen (2017), often emphasises the convenience it offers participants, who can more easily coordinate attendance with other personal and professional commitments. Another benefit is the possibility of more reflective ways of meeting, for instance because time passes between encounters or because presentations can be seen or read at leisure. Thus, asynchronicity has been seen as the best option to support participants subject to multiple time zones, clashing commitments, and less reliable infrastructures (Carr & Ludvigsen, 2017). However, without the nudge provided by the immediacy of live events, there have been concerns about engagement levels at asynchronous events, such as low attendance at Q&As following pre-recorded events (Raby & Madden, 2021) or where online discussions are dominated by a few prolific participants (Carr & Ludvigsen, 2017, p124). While noting that it is common in online communities to have many more ‘lurkers’ than contributors, the suggestion has been that multiple avenues for interaction be provided to meet the complex preferences of attendees (Carr & Ludvigsen, 2017).

Others have experimented with varying amounts of synchrony, such as adopting long time frames (up to a month) across which presentations are offered, early distribution of recorded keynotes leaving the common time slot open for discussion, and threaded forum discussions (Green, 1998; Lortie, 2020; Niner & Wassermann, 2021); there is even a proposal for a time-zoned “wave” of discussion, conceived as a continuous, relayed debate from one side of the dateline to the other (Ruddick, 2019). Importantly, while there are examples of video conferencing across the last few decades (eg Green, 1998; Waitt et al., 2012), many have felt that synchronous online conferences have only become a realistic possibility very recently, as broadband infrastructure and software has improved (Carr & Ludvigsen, 2017, p. 120). Among the lessons learned from recent experiments with synchronous conferences, particularly during the pandemic, is the danger of “screen fatigue” (Foramitti et al., 2021); and a sense that, in a constrained time-frame, technological glitches with tools like YouTube (live streaming) and Zoom (broadcasting), or screen sharing etc. become more disruptive (Rose et al., 2020).

Despite these promising forays into online possibilities, there remains a widely shared concern that one of the crucial functions of a conference – chance encounters and socializing at the margins of the program – are lost (Houghton et al., 2020, p559; Roos et al., 2020). To address concerns about the loss of “thick copresence” (Urry quoted in Higham et al., 2019 p616), Higham and colleagues have suggested an ambitious program of careful attention to the mobility practices of academics and how they are conditioned by the workplace imperatives of, what Hopkins et al. (2019) have termed ‘aeromobility’. Other approaches have emphasized experimentation with local-in-person hubs, or hybrid conferences, with participants still benefiting from face-to-face interactions but without the high carbon costs of international travel.^2^ Further options have been to turn to experimental technologies that can support looser engagement outside of the programmed sessions. For example, after research that found that social aspects are in fact the ones most valued by conference attendees, a team at MIT’s Sloan School of Management created an app, Minglr, which seeks to recreate the “coffee queue” encounter online by matching conference participants up with others they would like to speak to (Song et al., 2021). This is just one experiment among many with some online conferences now offering virtual “cafes” with tools such as SpatialChat, Wonder and Gather, or informal backchannels using Slack or Telegram (Rose et al., 2020). Indeed research has found that there *are* possibilities for the proverbial water cooler experience in more mainstream digital spaces, for example using the chat function in video conferencing software (Roos et al., 2020). Nevertheless, as Roos et al point out,

while the ease of setting up new webinars is an excellent opportunity for bringing new voices to the scientific community, this comes with the danger of online events being created with little thought and purpose, simply because everybody else is doing so. (2020, p5)

The current literature thus suggests that in our engagement with online conferencing as an ongoing experiment, we need to consider software, event design and planning as integral parts of the work that is to be done.

The UK-based Temporal Belongings Network has been running events on social aspects of time since 2011. From the outset, our unofficial motto has been “we don’t just talk about time and community, we experiment with them as well”. Thus we have always been interested in *how* we meet as researchers, including ways of challenging the largely passive nature of academic events (Bastian, 2014). Alongside this, the Lifetimes group in Oslo have been researching practices of synchronization across cultures and historical periods (Jordheim & Ytreberg, 2021), while the Waiting Times project has focused on the relations between time and care, particularly in healthcare contexts (Baraitser & Salisbury, 2020). Together, we saw the move to online as an opportunity to experiment with care-full synchronization practices. How might chronological synchronization based on UTC and time zones be augmented to include more experiential forms of synchronization? When looking at models that had been developed so far, we felt that there had already been exciting experiments with asynchronous delivery (NCN), multiple hubs (#Displacements) and repurposing well-known platforms (Twitter conferences).^3^ While we saw the benefits of asynchrony, we were also not fully convinced that making conference content always available is the only way to solve the problem of differing time zones and clashing commitments.^4^ As we know from research on people’s experiences of time squeeze, allocating time to non-essential activities can quickly become overwhelmed by pressures to manage the multiple schedules of other commitments (Southerton, 2003). We figured that the large gathering of a conference, with its concentrated pace, slight overstimulation, and intense social situation, would help participants to dedicate themselves to the event, rather than fitting it around other things. For all these reasons, we were interested in creating a concentrated “event time” that could produce a sense of co-presence.

## 3 What we did…

The Material Life of Time conference ran across a 49-hour period from the 15th to 18th March 2021. We had 418 registered attendees, and the event included 201 presenters, 45 panels, five keynotes, as well as workshops, roundtables, exhibitions, film screenings, and live music. In the sections below, we unpack three of the key areas that we considered in our planning for conviviality, namely how we created a shared time, a shared space and a shared sociality.

### 3.1 Convening Across Time Zones

As many have already experienced during the pandemic, online events cannot use the same schedules as in-person conferences and be inclusive for international participants. Prior to the pandemic, participants at in-person conferences were expected to adjust to the time zone of the event and deal with their jet lag as best they could.^5^ It is now easier to see that asking people to participate at ‘their 3am’ is unreasonable, particularly without the environmental cues of a new location that might offer some support to the weary international traveller. It is also much harder to participate online over the long periods of time that are customary for conferences: many run continuously from early morning to late into the night. Even so it is the synchrony that many credit with enabling conviviality and chance encounters, and which we wanted to retain. Our solution to these issues was to move to a more dispersed ‘time block’ structure. This approach built on a 2020 pilot event (Bastian, 2020), where we identified a one-hour block suitable for a synchronous meeting of our participants from North America, Western Europe and Eastern Australia (20.00-21.00 GMT). Using this as a keynote slot to anchor our timetable, we identified three further blocks which would allow attendees from at least two major continental regions to interact synchronously, while also having significant periods of downtime between sessions.

**Figure 1:**
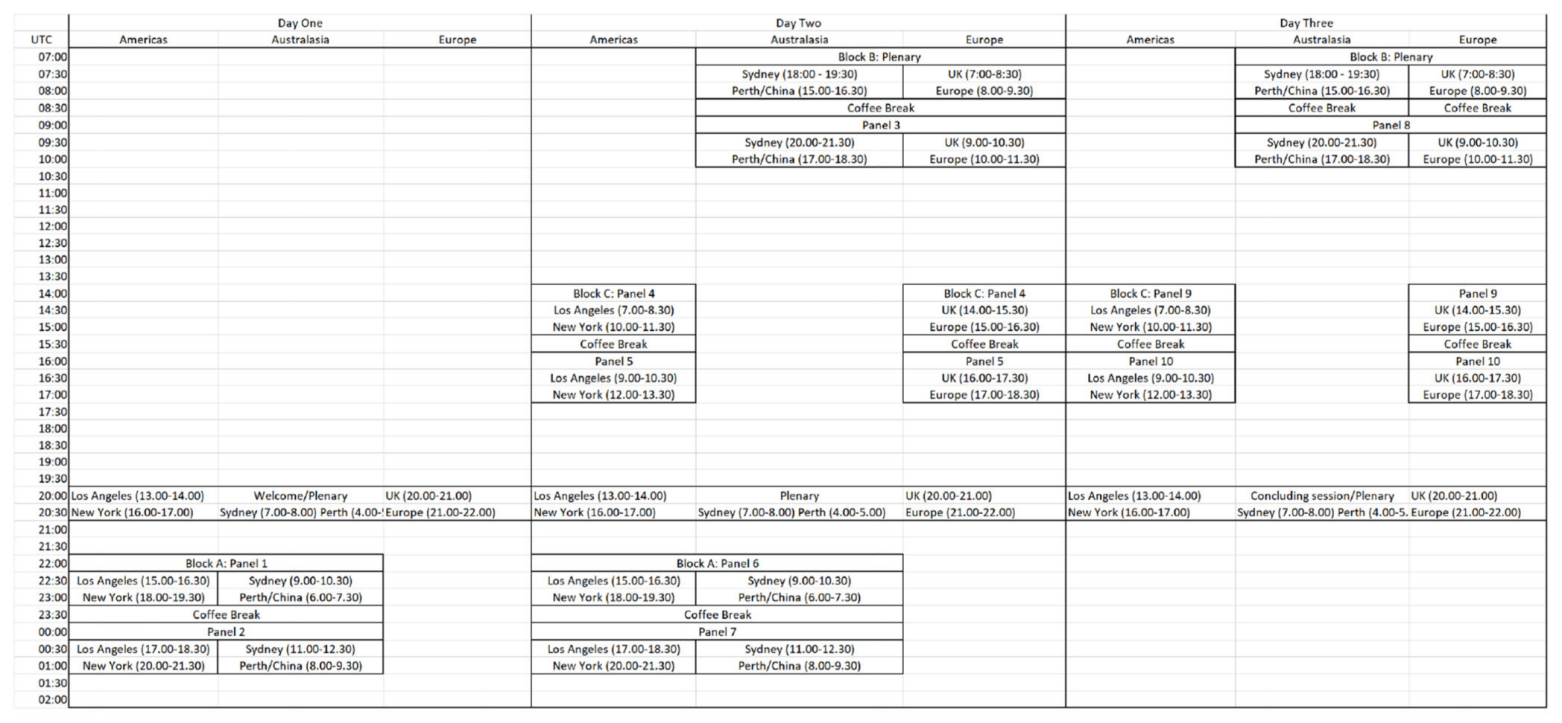
Skeleton schedule for The Material Life of Time

Initially focusing on the continents where the large majority of our members came from, we identified the following: Block A (22.00-01.30 GMT) for the Americas and Oceania; Block B (07.00-10.30 GMT) for Oceania and Europe; and Block C (14.00-17.30 GMT) for the Americas and Europe. Further, while our one-hour keynote block worked for the Western hemisphere and parts of the Eastern hemisphere (particularly Oceania), the first part of Block B was optimal for the Eastern hemisphere (Africa, Eastern Europe, Asia and Oceania). Given the latter would provide a key point of entry for attendees from these regions, we incorporated further keynote sessions in the first half of Block B to signal another significant gathering point (see Figure 1). Put together, any attendee around the world could expect to be able to attend at least one keynote session and one full block per conference day. Still, this schedule was certainly not a neutral one, and organisers wanting to centre different regions should identify blocks that are optimal for their target pools of attendees.^6^

**Figure 2:**
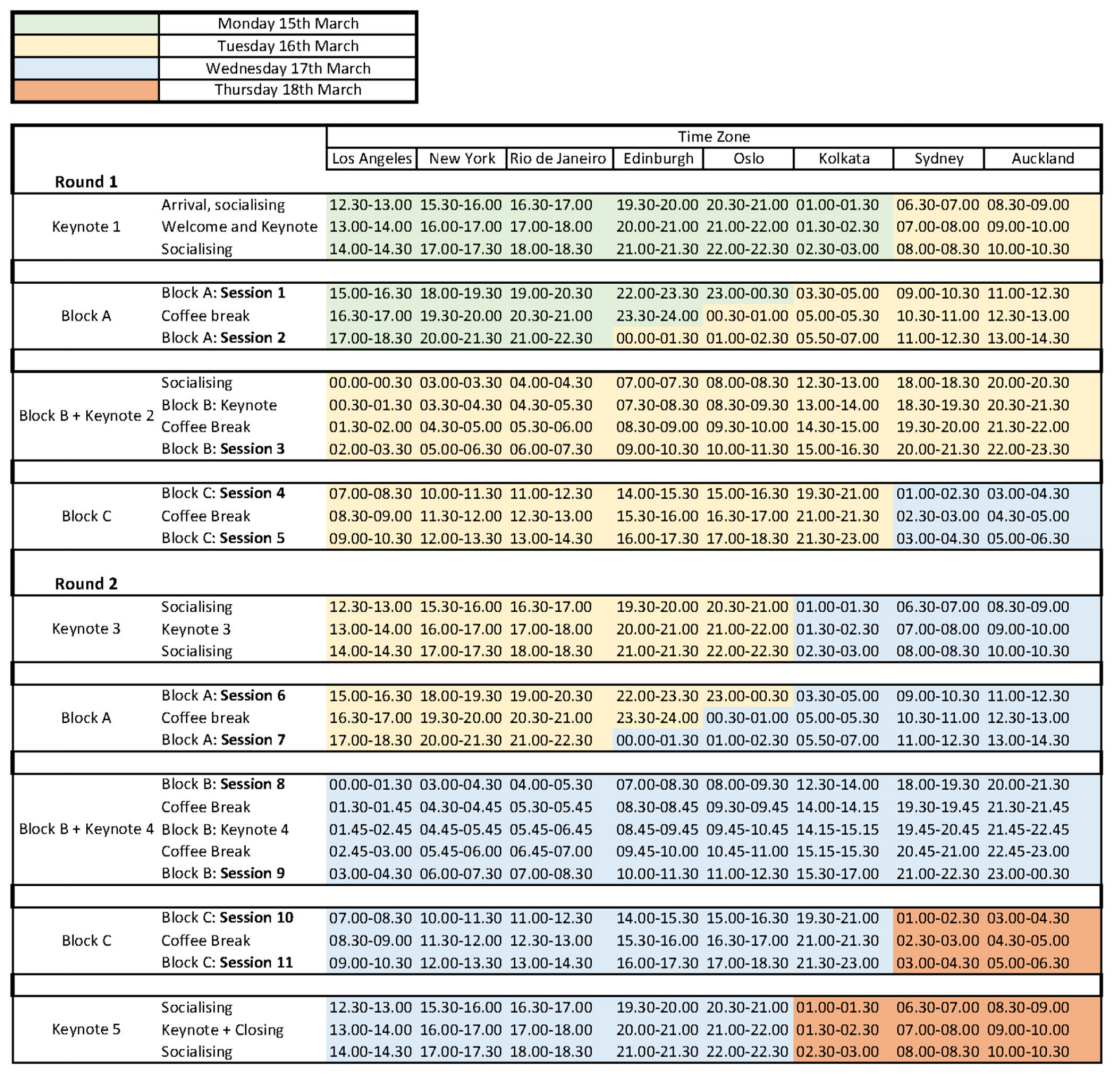
Colour-coded schedule for The Material Life of Time

Communicating this temporal structure became a design problem in and of itself. When we first announced the call for papers, we shared the ‘skeleton schedule’ seen in Figure 1, (and later on a colour-coded schedule in multiple time zones [see Figure 2]) so that attendees could see whether the timing would work for them while considering whether to submit a proposal. From the outset, this helped to demonstrate we were not ignoring the time zone problem, particularly to those often ill-served by European-based online events.

Aside from our block structure, many aspects of the temporal design remained similar to a regular in-person conference, with keynote sessions, panel sessions and coffee breaks. Presentations were shorter than normal with plenaries of half an hour, and panels of four papers of 15 minutes, and we doubled the typical 15-minute coffee break to 30 minutes. By making time zone compatibility our lead design requirement, however, we were pushed to include large breaks between blocks. Ideally, we imagined this would provide opportunities for attendees to spend more time in activities in the socialising spaces, as well as rest or take care of other responsibilities. Finally, we continued to incorporate an asynchronous element in line with our previous events, by recording all sessions and making them available for a month after the event for catch-up.

### 3.2 A *Place* to Meet Online

The next key issue for us was not so much the technical design of the event, but its spatial ‘feel’. That is, while we wanted to make sure the tech was appropriate and effective, our main criteria were that these tools create a feeling of freedom of movement and a spatial (in addition to the temporal) togetherness. While Zoom works well for presentations in terms of stability, it does not easily allow participants to move between breakout rooms as they wish, or choose who to speak to (Higham et al., 2019, p623). Having found options such as MIT’s open source Unhangout (https://unhangout.media.mit.edu/) where the design focused on peer-to-peer events, we looked for platforms that would allow our attendees to have more control over their movement within the online space. Our selected option, QiQoChat (https://qiqochat.com/), is described as a ‘social wrapper for Zoom’, and was originally built to support the participant driven meeting style known as Open Space Technology (Owen, 2008). Here, attendees can move at will between programmed zoom meetings, take notes collectively, chat and propose discussions with others using open zoom meetings in “The Gardens”. Crucially, the platform is also highly customisable, and our event was strongly underpinned by the services provided by QiQoChat for event design and tech support. In addition, we enrolled consultancy services from Improbable, a team of improvisers, theatre makers and conversation facilitators, who had been making use of the flexibility of the QiQoChat platform to design appealing and inclusive online events.^7^ Thus it was the combination of the tech and the design support that was crucial for us in moving from a virtual space to a welcoming *place*.^8^

**Figure 3:**
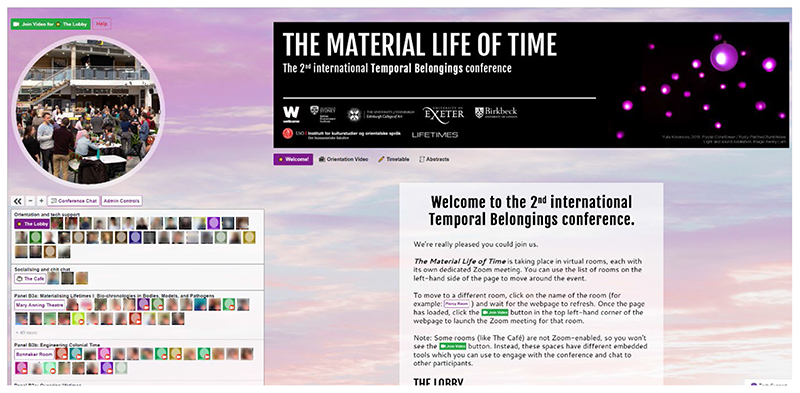
QiQoChat platform with landing page (Used with permission)

Within our QiQoChat event, we sought to replicate many of the familiar features of an in-person event, unlike a number of online event organisers who have expressed a desire to avoid the traditional conference model and explore the new possibilities of a shift to the digital (Houghton et al., 2020, p572; Robinson et al., 2020; Song et al., 2021). Given that so much else around the event would be new for attendees, we wanted to plug into shared knowledge of how conference spaces work to lend a feeling of familiarity and recognition. As seen in Figure 3, our landing page was set up as a virtual lobby, with relevant imagery and live support from QiQoChat and Improbable available. Parallel sessions were held in ‘rooms’ that we named after time-related figures, such as the Mary Anning Theatre and the Banneker Room. We also had a ‘café’ space which we will discuss in more detail below. Moreover, while at our previous in person conference we had had to be conservative with our room usage because of costs, our online platform allowed us to include a wider variety of spaces such as a gallery, a cinema, a games room (with collaborative puzzles) and a chill-out room (with the pandemic favourite WindowSwap). The latter two drew from examples of other QiQoChat events including those designed by Improbable, but we were also influenced by reading Harry Josephine Giles’ very helpful guide for autism-centred design of spaces.^9^

Even while we hoped that the platform would be intuitive, we anticipated that there would still be some need for participants to familiarize themselves with the spatial interfaces we had designed, including the QiQoChat platform and the SpatialChat tool in The Café. We thus offered one-on-one tutorials to all our keynote speakers; ran two training sessions, one for chairs and another for all participants; and we opened up the online space one week in advance for participants to explore on their own. For those who could not attend these orientation events, we also made a series of short videos providing a virtual tour of the site, the first when we sent out the call for papers for workshop organisers and others to use in their planning, and the second, more detailed one, a week prior to the event once the design had been finalised. Again, these activities emphasised for us that moving from online space to online *place* happened via a peopled online landscape with hands-on support and relaxed interaction, including throughout the event, most notably via the excellent work of our chairs.

### 3.3 Creating a Sense of Sociality

No matter how well the virtual space and temporality of an online conference is designed, concern about the inadequacy of socializing online has remained. Other commentators on greening academic practices have remarked upon the stark disparity between worries about a nice chat in a conference break versus the effects climate breakdown is having on lives, livelihoods and ecosystems right now (Houghton et al., 2020, p559; Robinson et al., 2020). Ironies aside, we suggest that devising appealing ways of meeting online can only be of benefit in galvanising more support for a significant shift to online engagement.

**Figure 4:**
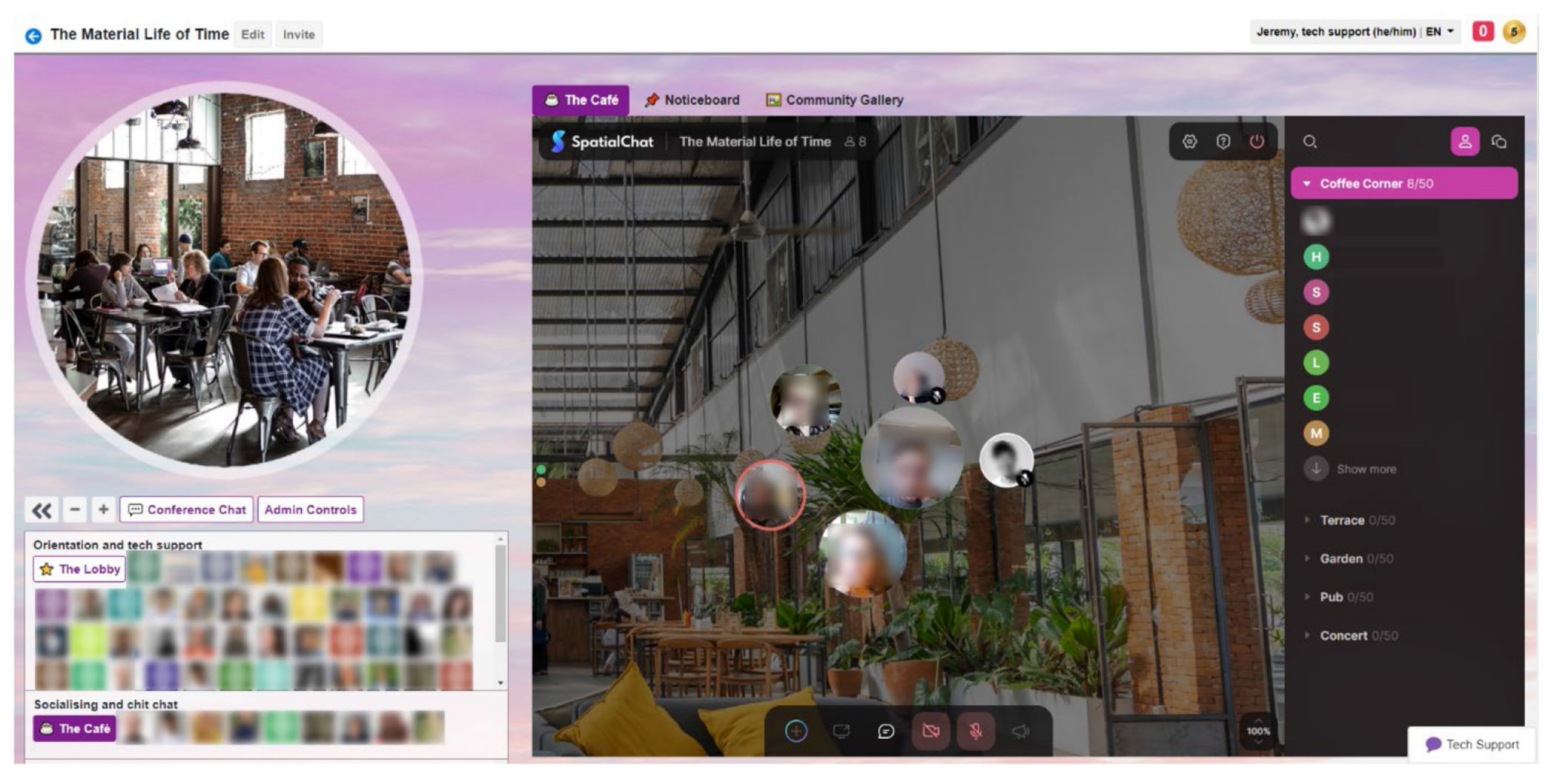
SpatialChat cafe at the conference (Used with permission)

**Figure 5:**
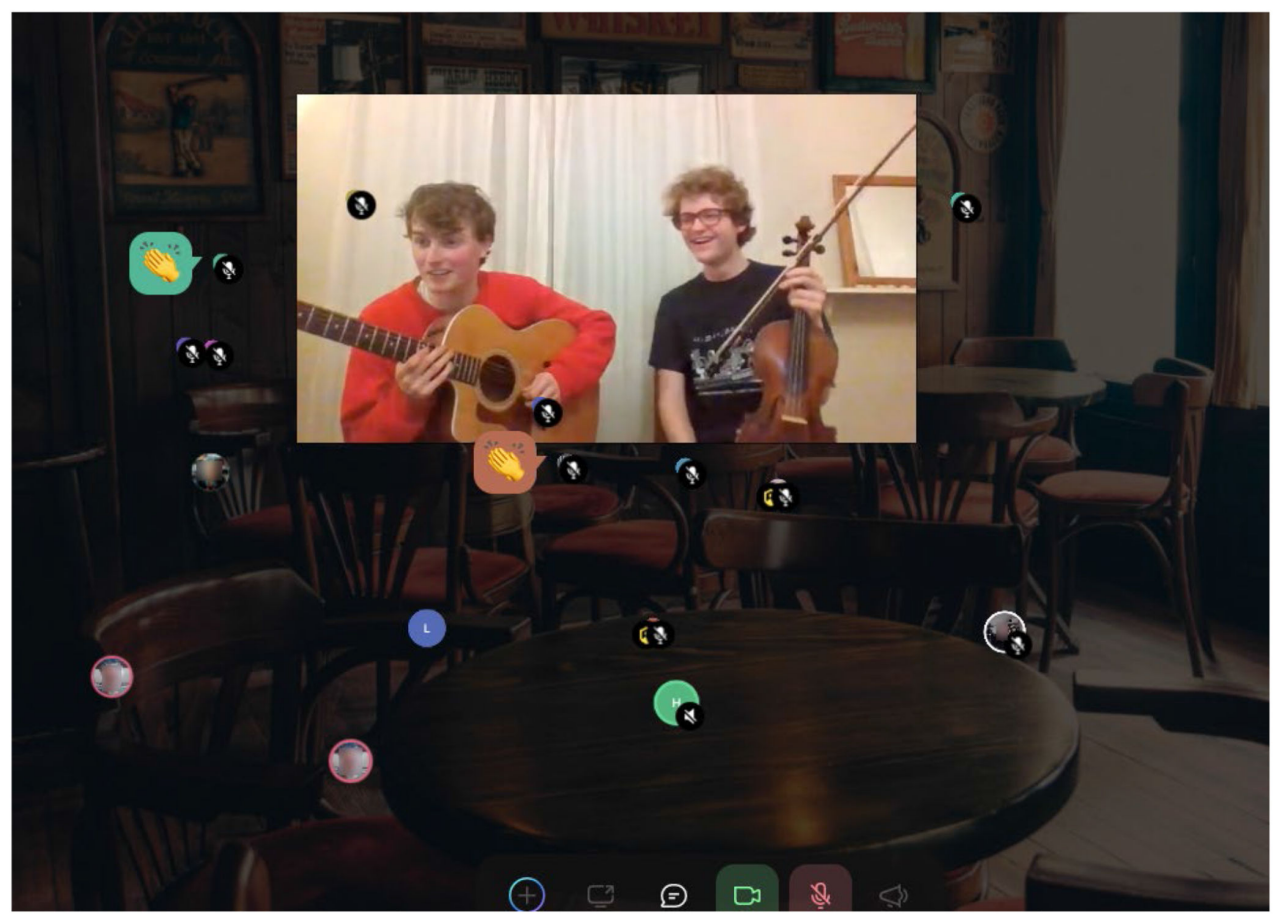
Jonathan Foster (right) and David Lennon (left). Members of the Edinburgh University Folk and Traditional Music Society at the conference Ceilidh (Used with permission)

**Figure 6:**
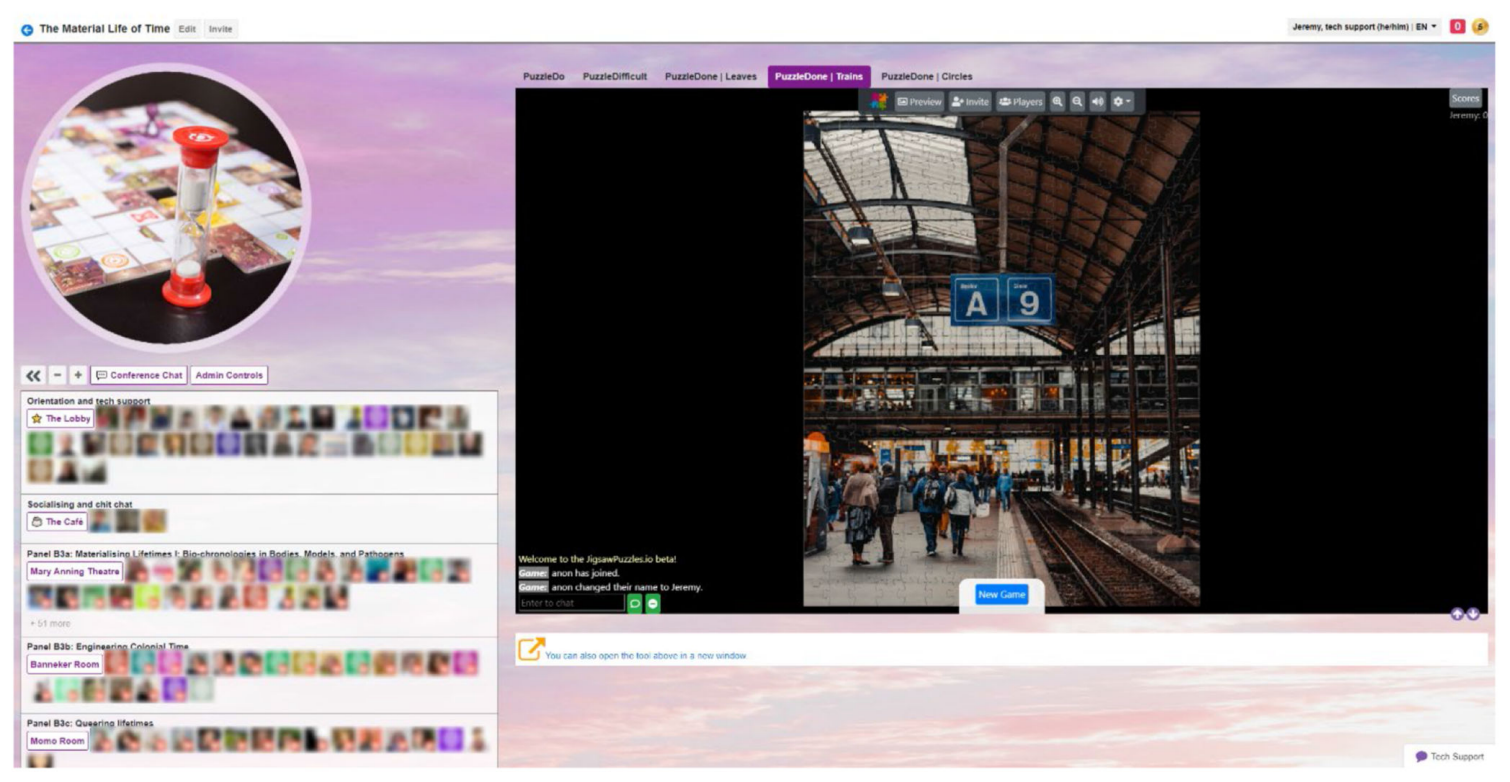
The Games Room at the Material Life of Time (Used with permission)

Our social design was subtle in some ways. For example, QiQoChat is designed so that on the RSVP page, attendees can start getting a sense of who is coming as profiles are updated with a bio and photo. When entering the event, they can see the moving avatars of others – as some attend sessions, others hang out in the lobby, and still more visit the social spaces (left panel in Figure 3). Thus, even without participating in the dedicated social activities, attendees already had a sense of being with others throughout the event. After a Zoom meeting finished, for example, rather than being abruptly thrown out, attendees could still see others milling about or other sessions still ongoing. More obviously in terms of social design, we created a café room where we made use of a multi-roomed networking tool – SpatialChat. Here, attendees could move freely around spaces that we styled as a café, terrace, garden, pub and concert space, while video-chatting with those nearby (Figure 4). The SpatialChat functionality means that the closer participants are to one another, the louder the volume of their audio becomes, allowing them to move their video avatar around the virtual room to selectively engage in conversations with different people. Participants were prompted to “go for a coffee” at the end of each session by our chairs, and we also organized activities such as an early career mixer and live concerts after keynote slots, including a traditional Ceilidh (Figure 5). In addition, the Games Room (Figure 6) had a chat feature available whilst completing puzzles, and there was also a general chat tool available via the open source RocketChat.

As we noted in relation to producing a sense of place, the tech platform only worked when there were people carefully attending to the task of building a shared sociality. Thus, the important work of welcoming and connecting took place in our scheduled Zoom sessions too. As with our in-person Temporal Belongings events, rather than launching straight into content delivery, we took a moment to say hello to people arriving and for them to communicate something about themselves to each other. Asking for responses in the chat to questions such as “what is the colour of the sky where you are?” aimed to help attendees feel more actively involved, moving from audience to participants, and get a better sense of “who is in the room”. This time was also used by some participants to acknowledge the traditional custodians of their particular location, raising a political question about what time and space is inhabited within the digital and how indigenous and settler relationships are recognised within these environments. Further, given that attendees already had to pass through the registration process to access the Zoom calls, we did not need to lock down participation for fear of Zoombombing and so chat, video and live questions were possible, with people encouraged to message publicly or privately throughout the panels.

## 4 How it went…

To understand where we were successful in fostering conviviality and where we needed to rethink our approach following the event, we drew on a variety of materials. This included our own observations and experiences as organizers and participants at the conference, and the feedback we received in person, over e-mail and through more direct correspondence after the event. We held debriefings with the organizing committee to consolidate lessons learned. In addition, we surveyed all registered attendees. The survey comprised 16 questions which asked attendees to rate their overall satisfaction on a scale from 1-10, state their career stage and geographical position when attending the conference; what parts of the interface they had used during the conference, some multiple choice responses using a 5-point Likert scale and six more open-ended responses. The open questions asked attendees to share their impressions of the temporal, spatial, social and academic aspects of the conference. We received 96 responses, a 23% response rate. Our analysis draws out themes that arose from all of these sources, with particular focus on the success (or not) of our experiments with online conviviality and lessons for other event organisers. Where data is available we have supported our reflections with quotes from free-text responses and/or descriptive statistics from the survey. This has allowed us to show some general trends from our participants, as well as useful individual insights from the organisers and attendees. Note that as humanities scholars we did not conduct more detailed analysis of the survey responses, such as unpacking the survey results according to different characteristics (which we generally did not ask for). However, we do indicate career stage and location at continent level for all respondents quoted directly to help provide more context.

### 4.1 The Online Experience, convivial or not?

Perhaps the most substantive goal of our conference design was to build an online experience which would provide a meaningful sense of coming together ‘at’ an event, and allow for participant interaction and relationship building. This is the most complex and contested issue raised by this event. When asked for their opinion overall, attendees rated their satisfaction very highly (in fact slightly higher than our previous in person event with an average response of 8.5/10 versus 8.4/10). Nonetheless, when asked to reflect on whether future events should continue to be online, 46% of respondents expressed concerns about losing the benefits of physical presence within their free text responses. A handful employed language like “no substitute” (later career tenured, North America); “nothing like [it]” (student, Europe); or “irreplacable” (student, North America). To the extent respondents detailed exactly what these perceived benefits were, they included: (informal) networking opportunities, body language and spontaneity of communication, the entertainment value of physical presentations, and the joy of “spending a few nights in a new city and making friends over beers” (early career non-tenured, Europe). At the same time, however, others lauded the online format for creating a “sense of focused interest that is much rarer at larger academic conferences” (student, North America), “a sense of intimacy” (early career non-tenured, North America) deriving from watching broadcasts from presenters’ homes, and an atmosphere where “much of the usual hierarchies were less present which created a very open and vibrant discussion” (student, Europe). Ambivalence was thus the majority view among the free texts responses to this question (58%). The texture of the expressions of ambivalence suggested that attendees were committed to the practical and emotional labour of acquainting themselves with an online format, and of weighing the losses associated with this shift in academic culture and daily life.

### 4.2 Many participants had an experience of shared presence

We received feedback both in the survey and via informal channels about the sense of ‘being together’ that the conference managed to evoke. A repeated point of feedback after the event was that participants had felt like they had actually been ‘at something’, that they had ‘gone to something’, in contrast to other online events which seemed to fade from memory. As one respondent wrote:

I loved the experience and was amazed that you had managed to create a sense of an actual event. Even when there was nothing happening in my time zone, I had a sense of others, elsewhere, at the event. And during our sessions I had a feel of others, elsewhere, asleep. It was quite remarkable to actually “feel” the whole thing going on, even in the background. It didn't feel like a set of separate links to separate talks. (Later-career tenured, Oceania)

Part of this could be attributed to the spatial design, with its re-creation of the rooms and theatres familiar from in person conferences and the presence of avatars moving around the space. The liveness of the event was another factor that was mentioned. One respondent appreciated the uncanny experience of participating simultaneously with people on multiple continents; another, the “non-stop” effect of a program catering to different time zones (Later career tenured, Europe). A third example:

It was a new experience for me to chat with people from other parts of the globe in real time: very exciting one. Although it did mean asynchronicity in the way we perceived the time of the day, some with coffee mugs, others with wine glasses: it sets the mood. But the mixture of different time-zones had its equalizing effect. At offline events there is a dominant time zone (that of the organizers) that sets others with jet lags to a challenge. (Early-career tenured, Europe)

Even so, others noted that the distribution of the blocks of sessions left a comfortable amount of rest in between. The success with creating a sense of ‘eventness’, or ‘virtual buzz’ as Hracs and Pinch (2020, p21) have discussed, was credited by various respondents who drew it out to the choice of having live, as opposed to pre-recorded, talks; the organization of the time blocks, and attention to time zone plurality (a particular concern of the Australian members of the organizing committee, who have experienced their fair share of time zone discrimination);^10^ and “small tricks” like starting off keynotes with icebreaker questions (“What did you eat most recently?”), which helped attendees to feel together in the midst of their temporal similarities and differences.

### 4.3 Mixed results from dedicated socializing spaces

How the virtual social spaces would work was one of the things we as organizers were most curious about. These spaces were not as frequented as the formal presentations, or the keynotes which were the most highly attended. Of our survey respondents, 100% participated in Zoom sessions, 51% participated in The Café, 32% in the Gallery and/or Cinema, and 15% tried the Games Room and/or Chill Out Room. These numbers accord with previous research, where about half the attendees attended online social activities (Raby & Madden, 2021). But while the number of attendees may have been reduced in the social spaces, there were many reports of real interaction taking place. After our first keynote, the speaker moved to the Café, and much like at a real event, audience members were milling around them in a circle, some listening and others waiting to get a word in. Another organizer logged in to the space to find two senior scholars playfully bouncing around the social space in SpatialChat, where those present are represented by a movable ball. In responses to our question on ‘spatial’ experiences, which explicitly asked for feedback on SpatialChat, ambivalence to the Café was again prominent. One respondent found the space weirdly prescient (“I had this sense like we were watching something that in years to come will be far less clunky” [Later career tenured, Oceania]), another “loved, loved, loved the live music” (Later career, non-tenured, North America). Overall 18/72 of respondents (25%) were clearly positive. Some found the interface unwieldy or disorienting, while a significant number (15/72 [21%]) expressed feeling intimated, shy, awkward or uncomfortable. Most of them felt discomfort around socializing with strangers or striking up random conversations: Only two voiced discomforts around trying to socialize with people they already knew in the spaces. How much of the discomfort was medium specific, as opposed to the awkwardness of conferences in general, was also remarked upon. As one respondent wrote (we think ironically):

Spatial Chat in the cafe successfully replicated and amplified the social anxiety of being at an actual conference so that was a treat. (Non-academic, Europe)

A couple of events in the social spaces were singled out for praise: One was the live concerts, which enabled people to be in the space with the anonymity of an audience member. Another was the early career mixer, which was actively hosted, making it easier for participants to familiarize themselves with the new technology. And while the interface was hard for many when striking up conversations with strangers, others suggested it worked well for them when catching up with people they already knew, or who they met in one of the sessions and arranged to meet for a conversation afterwards.

Whether this array of experiences differs from real-world encounters is worth asking. Arguably, the experience of awkwardness on the platform is a sign that the social situation was working, that something interpersonal was happening – navigating awkwardness is of course a mainstay of conference survival tactics anywhere. In general, familiarization seems to be key: One respondent wondered whether the virtual socializing came more naturally to video game players – suggesting something about how academic online culture may evolve as people habituate themselves to living their lives online. Our training sessions were also credited with making the space seem easy to navigate, helping people over the phase of any initial discomfort and leading to positive feedback around the friendly and knowledgeable chairing. Interestingly, some respondents pointed to the unstructured time pre- and post-Zoom seminar as being of similar, if not more, importance for networking as The Café. This unstructured time primarily took the form of conversations between panel members while setting up, and with early arrivals. Enabling these interactions is thus an important, if overlooked, ‘corridor’ type space which is not currently well supported by automatically keeping attendees in waiting rooms, or closing meetings abruptly at the end of a session. The public and private chat functions of a Zoom room was another cited example of an arena for socialization that was not really designated as such.

### 4.4 “I felt it was so generous” – the *je ne sais quoi* of a good vibe

The art of hosting proved to be just as much of a social responsibility online as it is in a physical location, if not more. Given that online social norms are subject to discussion and play, and that different online setups will require different norms, continually paying attention to both the mechanics and atmospheres of connection are vital. This includes providing opportunities for attendees to connect, while also being clear about *how* they can interact and when. For example, we realised that we need to be much more proactive with The Café space, including providing explicit opportunities for attendees to learn how to use it, providing conversation starters through organised topic sessions, and having dedicated hosts in the space to answer questions and provide a friendly point of contact. During the sessions themselves, it proved invaluable to prioritise ways for attendees to ‘be in the room’ together before jumping straight into content dissemination. This meant that even those who never went near The Café still interacted with others. Finally, hosts have significant opportunities to set the mood in the way that they open online sessions. The conference hosts were credited with having a “warm and welcoming presence” that created a “lovely atmosphere” (Later career tenured, Oceania), and we believe these personal touches of speaking directly to attendees when they first arrive (rather than silence until formal proceedings start); signposting that participants are all engaging in a shared experiment and normalising ‘mistakes’; while also having opportunities to share what is happening where they are (rather than being faceless audience members); worked particularly well. This active ‘hosting’ work suggests that creating new habits and forms of community online is not just a matter of planning, design and technology, but about personal touches that aid connection.

### 4.5 Connections made

These exercises in conviviality were not just about enabling a sense of freedom and enjoyment at the event, but also to address concerns about informal networking, and so, in this section, we look more closely at how successful we were at recreating this aspect most often credited to ‘the coffee break’. That is, did the sense of shared presence felt by attendees translate into connections that would be taken forward? First, we asked survey respondents to compare the number of new connections they made to those they make at a regular conference. The results showed that 30% made connections far below average, 41% were somewhat below average and the remaining 29% were average or higher. This means that 71% felt they made fewer connections than usual (see Table 1). At the same time, however, the large majority of participants (70%) did make some connections. In and of itself, this challenges the expectation that informal networking is nearly impossible to replicate online.

Perhaps one of our most welcome findings was that when asked about whether participants felt they would stay in touch with these connections as they usually would, 55% said they would stay in contact as much as usual, with 8% suggesting that they would stay in touch more or much more than usual (see Table 2). In fact, only 11% of respondents indicated that they would stay in touch with new connections much less than they usually would. This suggests that even though there were fewer connections made, the majority (63%) felt that these connections were at least as strong as they would have been from an in-person conference.

### 4.6 Online conferences offer the convenience, but also the traps, of multitasking

Our demonstration that networking is feasible is complicated, however, by the fact that our event took place during the ongoing Covid-19 pandemic where academic workloads and caring responsibilities have increased significantly for many. Although, at the same time, others have had more time on their hands due to furlough or retrenchments. Further, in order to take advantage of a beneficial overlap of time zones and daylight saving time between key regions, which supported wider windows of synchronicity, the conference occurred in the suboptimal academic time of March (with some attendees in the midst of start of term or assessment seasons). In this and the next section then, we reflect on the challenges presented by our focus on synchronicity within a pandemic.

Recognising that networking could be affected by participants’ availability, we asked survey respondents for their sense of how busy they were and what their attendance was like compared to usual (see Tables 3 and 4). Indeed, 57% of respondents described themselves as very busy or extremely busy with other responsibilities during the conference. This may have contributed to the fact that 68% said they attended the conference somewhat less or much less than they would at a typical in-person event. This leads to a conundrum in the planning of online conferences: How do we respond to the paradox that the informal networking that many say they miss from online events, is also the easiest part to skip when having to make hard decisions over where best to spend one’s time?

Indeed, a challenge that was remarked upon by at least 10 respondents to the survey in free text questions had to do with making time. Some respondents expressed regret that they did not sufficiently “block off” the days on their schedules; others remarked that participating online made it impossible to take leave of their other commitments, whether it be teaching, other obligations at a home institution, or havoc in the home office. For example:

Very hard to focus on fascinating ideas when looking for the ‘emergency’ lost book for online school, cook, manage household problems, deal with the meter reader, etc. (Later-career tenured, North America)

This suggests that making a sense of shared, active presence requires not only a good setup for the conference, but planning for the fact that participants at an online conference are not transplanted to a different location where they are regarded by both themselves, and those with claims on their time, as being ‘away’ (although Hopkins et al [2019] remind us that this ‘away’ is not always carefree). In fact, one of our key concerns is that the rise in online events – driven by the pandemic with the increase in precarity and responsibilities pciated – has led to a de facto understanding that online events should be fitted in around other activities (Houghton et al., 2020). We believe this view needs to be robustly challenged, in part because it risks creating a vicious cycle where attendees feel unable to make time for informal interaction, online events are then seen as unable to ‘replicate the coffee break’, and individuals, universities and organisations are then less committed to giving these events the time they need to really work.^11^

Action is clearly needed both at an individual and a collective level. It may be useful for conference organisers to bring the issue up explicitly and suggest to attendees that they consider in advance how they might protect some availability for both the formal and informal activities. This can be difficult to do sensitively, as many working in academia were already overstretched prior to the pandemic. Thus, collective action is also needed to ensure that our institutions send clear messages that staff and students should not be expected to hop into meetings or take care of administrative tasks when they are attending online events. For organisers, a core element of facilitating this change is thoughtful timetabling, carried out in advance, and clearly communicated to participants. This might start right at the outset, when a call for papers is announced, providing not only the dates of the event but more detailed information about the format and the time blocks that will be used. Importantly, this information also enables participants in other parts of the world to know whether or not their temporal situation is being considered in the planning of the event, and so to make a more informed decision about attendance with a fuller sense of what it will require of, and mean for, them. As discussed below, this communication work is not without its challenges, something that we learnt about as we went.

### 4.7 Communicating time zone plurality is key

In many situations in everyday life, synchronies are already baked in, with shared standard work hours and other institutional pacemakers (schools, transport schedules etc) guiding our time (Mulíček et al., 2015). While these standards certainly do not work for everyone, they are widely understood. In our case, making time was not only about attendees finding spots in their day to attend, it was also about making a new social pacemaker, if temporary, in the form of the conference itself. Our event needed to pull our attendees into a rhythm outside their everyday norms in order to synchronise across the globe. We learned that this is even more complicated than we initially thought. For example, although we took great care in the planning and design of the schedule spanning many time zones, we were less attentive initially to how the schedule would be communicated to all attendees. While the pandemic could be said to have encouraged greater time zone literacy, we made fairly simple mistakes early on, such as advertising the event as occurring 15-17th March as it did in Europe, when in Australia it was the 16-18th March. We also found that any simple discussion of conference ‘days’ would not work, since ‘day one’ started in the evening for Europe and early morning the next calendar day for Australia. Here we adopted the term ‘rounds’ instead, with each round consisting of the keynote session and the three session blocks A, B and C. This was used at the conference to welcome attendees to the first and second rounds of our event. Understandably, in our feedback, we received comments in the free text sections about the schedule being daunting and intimidating, even while many appreciated the attention to temporal inclusivity. As one survey respondent wrote:

I had to keep asking smarter friends what day something would actually be on in my timezone. (Later career-tenured, Oceania)

Our experience thus suggests that planning a schedule across time zones and communicating the schedule are separate tasks, and that both require care and attention. This might even include inventing event-specific terms for the blocks of time that make most sense to the particular design utilised. This is particularly true because our experience of planning this event suggested that affordable bespoke software that supports planning and scheduling across time zones is hard to come by, and is often only available as features on clunky, expensive event platforms.

## 5 Final Thoughts

As we have sought to demonstrate in this article, cultivating a convivial online conference requires close attention to the temporal, spatial, and social elements of the event, from the outset of planning right the way up to that final casual conversation after the virtual ceilidh. When this is done, it is possible for online conferences to create the feeling of an ‘event’, a moment in time in which individuals have come together to share and connect. This emphasises *time* as a medium through which conviviality is produced – an ‘environment’ that is usually taken for granted and can remain neglected in relation to designing the spatial. The online format forced to the surface the need to organize, elicit, host, plan and take care of people’s temporal experiences, and this brought a new layer of conviviality to the conference experience in which participants felt ‘held in time’ by the conference itself. The well-worn customs and traditions of how to contain time in spatial ways (in plenary lecture halls, break out rooms, around the coffee machine, in social spaces after the conference has finished for the day), had to be rethought for an online event, and we found it was through the conscious containing of time in each element of the design that participants were enabled to feel they were part of something that accumulated *over* time to become, perhaps retrospectively, an event.

We achieved some measure of this through a mostly synchronous conference with a carefully planned online platform. But we also took advantage of the online format to allow the event to linger via catch up recordings. Other elements of the temporal planning that we thought worked well included the intricate organization of the conference time zones, and the attention to creating moments within the event for people to share their temporal situation and so experience the sense of being with others in a time that is both shared and not. ‘Making time’, in terms of communicating a program across time zones remains a challenge in need of creative experimentation. Visual aids offer some help. If available, software that translates all sessions into each participant’s time zone can be invaluable, although it may also disrupt a fuller sense of the multiple ways in which the event is being experienced by others, and so undermine the sense of a collective event in shared time.

The biggest temporal challenge we see, however, relates to the capacity of participants to ‘make time’ *for* the event in the same way that they would for an in-person conference. Clear planning and communication will go some way towards this goal, but it will ultimately also require cultural and institutional changes. Here, we issue a specific call for concerted efforts to question the assumption that online events should fit around other activities, and for inventive responses for how to solve the currently tight interconnection between spatial distance and freed up time. In particular we note Etzion et al.’s (2021, p3) point about a key feature of the conference being its very nature as a sometimes hedonic ‘routine-disrupter’ which enables an extrication from usual commitments, or acts as a jumping off point for a holiday or excursion which can be added on more easily since one is already ‘away’ (see also Hopkins et al., 2019). This suggests that we must not underestimate the role conferences play in shifting overworked academics between temporal modalities, and experiment with how these modalities could be activated otherwise.

The QiQoChat platform was at the heart of our effort to curate a distinctive *space* for this conference, which in turn was essential to the sense that it was a real event and that participants had in some sense ‘gone somewhere’. While a list of Zoom room links for each session might have coordinated people to the various presentations just as effectively, it would not have enabled this emplaced sense, nor would it have provided a sense of the broader community (in the form of their avatars listed in various rooms), or the various informal spaces we used. While it was not surprising that the informal spaces were at least as important for the convivial atmosphere as the formal settings, we were surprised by *where* sociality emerged: Conference attendees did not only socialize in the designated Café, but made use of whatever liminal spaces they could find – the Zoom chat, the preliminary wait in a Zoom room, the chat in the Games Room. Having multiple spaces of this sort, and using Spatial Chat in at least some of them, was also crucial to allowing more organic, less hierarchical, connections to emerge – the kind of conviviality which is more than enjoyment by including both freedom and interdependence as well. As anyone who has tried to socialise in a large group in a Zoom room knows, only one person can generally be heard at a time and discussions tend to be dominated by a small group. Creating alternative spaces, where people can interact in small groups, can be off to the side, or can be entirely unseen, is a vital part of making these kinds of social experiences more open and inclusive, and so often more able to develop into more substantial and lasting connections. However, our conference would have been enhanced by additional planning and instruction to familiarize participants with these interfaces, to invite everyone into these spaces, and to create effective icebreakers. Here, we would recommend that conference organisers investigate ways of opening up the platforms they use as much as possible, to allow for the convivial freedom of movement while mitigating the risks. When choosing between platforms there are thus opportunities to think beyond concerns for stability, and to ask how participants will be able to meet when and where they want.

Managing these temporal and spatial elements effectively is necessary for creating a convivial online event, but such considerations are not on their own sufficient. The inter-personal *social* work of the host, or team of hosts, aided by volunteers, panel chairs, tech support, and many others remains vital. We worked to cultivate an open and inclusive feeling to the event, not only in planning but in the real-time running of things. In the online format, this inter-personal work was spread across a number of time zones, and interfaces, creating a sense of a distributed community of hosts, with colleagues in Australia awake and working on behalf of the conference while others slept in Europe for instance; or colleagues being available in the lobby or cafe while others chaired a keynote. This social work requires significant specialist skills. One member of our team (Bastian) has a long-standing interest in creating inclusive, interactive, spaces for learning and research and took on the bulk of this labour in a thoughtful and dedicated way. The event would not have been possible without her care and attention. But it also benefited immensely from input from many others. The bespoke virtual conference organizing services of Improbable, helped us in creating a concept, both organizationally and design-wise, that would deliver on our goal of sociable, virtual co-presence across time zones.

Reflecting on our own experiences, much of the extra labour from this conference came in the planning stages, where we needed to invent new ways of working, and identify and build new relationships with our tech and support teams. Committing to collectively sharing our learning from these processes is thus important for providing guides for others looking to experiment. While every event will be tailored for the specific needs of the community being brought together, we hope that our experiences will prompt new avenues for consideration. Building new ‘templates’ for academic events, which have remained largely unchanged for centuries, may thus help embed new familiarities and skills that would enable those who perhaps feel less confident or motivated by the design side of event organisation to still run enjoyable and convivial online meetings drawing from a new ‘tool box’ that we hope to contribute to with this article.

As researchers who work in time studies, our work pays particular attention to the temporal in situations where it can easily be overlooked. As a consequence, we were interested in designing and hosting an event that emphasised how sharing and containing time could produce conviviality, rather than relying only on spatial contact and welcoming configurations of space and place. In order not to repeat the exclusions of in-person events, however, careful planning and hosting is required, including creating new environments online that includes the elements that make it worth ‘giving up’ time to attend – chance encounters, unexpected connections, the pleasures of learning and coming together to form a temporary community, making lasting links. For this to happen, attention needs to be paid to how time is not simply managed but how it is shared.

This sharing includes creating a regular feedback loop from lessons learnt at innovative online conference practices into the future planning of events, taking heed of the need for attendees to be included in the planning so that numerous forms of exclusion are not repeated – from time zone exclusion, through to those exclusions that online formats exacerbate for some individuals. In addition, it is easy to stop short of a radical reshaping of conference formats when using software that produces specific configurations of space and time that often emerge from the corporate sphere. In our experience, working with QiQoChat, which is driven by a deep commitment to support dialogue and peace work, as well as Improbable, with their background in the theatre, performance, participatory democracy, and both with their commitment to Open Space, went some way to mitigating the effects of software choices on solidifying new norms for digital events.

In sum, we have suggested that ‘the coffee break’ problem can be tackled with a close attention to the elements key to these vibrant conference moments: temporal infrastructures that create a sense of togetherness; freedom of movement that enables participants to make decisions beyond whether they join or exit a Zoom meeting, and to instead form serendipitous connections; and a sense of place which is friendly, warm and welcoming. We demonstrated that even in a pandemic context, where many of our participants were very busy, they *were* able to make new connections, and that while they might have been less numerous, for most they were just as strong. Most importantly, we showed that when making efforts to change how communities interact it is not enough to only change the spaces within which we meet, we must also change our time.

## Figures and Tables

**Table 1:** Connections Made [Did you make connections with new people at the event (compared to your average experience at an in-person event)?] n = 93 respondents

*Response*	*No. of respondents (Percentage of responses)*
Far above average	1 (1%)
Somewhat above average	9 (10%)
Average	17 (18%)
Somewhat below average	38 (41%)
Far below average	28 (30%)

**Table 2:** Strength of Connections [How likely it is that you will stay in touch with any of the new people you've connected with from the conference (compared to your average experience at an in-person event?] n = 92 respondents

*Response*	*No. of respondents (Percentage of responses)*
Much more than 1 usually would	2 (2%)
More than 1 usually would	5 (5%)
Same as 1 usually would	51 (55%)
Less than 1 usually would	24 (26%)
Much less than 1 usually would	10 (11%)

**Table 3:** Busyness [How busy were you with other responsibilities during the conference?] n = 94 respondents

*Response*	*No. of respondents (Percentage of responses)*
Extremely busy	26 (28%)
Very busy	28 (30%)
Moderately busy	30 (32%)
A little busy	8 (8%)
Not busy at all	2 (2%)

**Table 4:** Attendance [What was your conference attendance like compared to your attendance at a typical in person conference?] n = 93 respondents

*Response*	*No. of respondents (Percentage of responses)*
Much more	0 (0%)
Somewhat more	6 (6%)
About the same	24 (26%)
Somewhat less	41 (44%)
Much less	22 (24%)

## Footnotes

1 Our use of ‘convivial’ has something of the flavor of Ivan Illich’s *Tools for Conviviality* (1973), which emphasizes freedom amid interdependence, as well as enjoyable encounters, in addition to the half-century long history of efforts, in activism, the humanities, the arts and associated public spheres, to devise conscious forms of communing in the face of the ecological crises wrought by fossil-fueled societies.

2 The AAG Regions Connect initiative in October 2021 being one example, but see also Martin, 2022; Padian, 2018; 2020.

3 Twitter conferences appear to have begun in 2015 with the first World Seabird Twitter Conference (#wstc1) and the Royal Society of Chemistry’s Twitter Poster conference (#RSCAnalyticalPoster). See also this recent analysis of Twitter as a conference backchannel at the 2018 AAG (Fekete & Haffner, 2019).

4 Notably, we have always included asynchronous online elements to enable wider participation, providing audio recordings and slides of talks on our website after each event with space for comments, somewhat like the NCN model. Interestingly, we did not conceive of this as an extension of the conference, but as supplying resources subsequent to the main event.

5 For an in-depth study of how academics at the remote University of Otago balance the career-building imperative to meet physically, and thus fly, with the imperative to fly less, seizing some of the opportunities that virtual meetings offer, see (Higham et al., 2019).

6 See for example the design of the EPIC2020 conference which also used three global zones. https://2020.epicpeople.org/pangaea/index.html

7 See https://www.improbable.co.uk/

8 For an alternative perspective on the ‘places’ produced by Zoom from a geographical perspective see (Hay, 2020).

9 See https://harrviosephine.com/portfolio/chill-out-corner/ Thank you to Fred Carter for bringing this to our attention.

10 Australians and New Zealanders, as well as others very far away from the metropole, are at multiple disadvantages in discussions around the climate impact of conferences, with some suggesting that they should only be allowed to attend European conferences very sparingly (Desiere, 2016), and yet finding it hard to attend large conferences, even online, due to antisocial hours.

11 We should note that some respondents saw the ability to multitask during online events as an asset. Participation was seen to require less of a commitment, and by not engaging so intensively, these respondents found themselves less fatigued than at an in-person conference.
